# Recurrent Symptomatic Severe Hyponatremia in Craniopharyngioma

**DOI:** 10.7759/cureus.79110

**Published:** 2025-02-16

**Authors:** Sumaiyah Fatima, Ram Pathak

**Affiliations:** 1 Internal Medicine, Marshfield Clinic Health System, Marshfield, USA; 2 Endocrinology, Marshfield Clinic Health System, Marshfield, USA

**Keywords:** craniopharyngioma, desmopressin, hyponatremia, pituitary tumor, syndrome of inappropriate secretion of antidiuretic hormone (siadh)

## Abstract

This is a case of a 20-year-old male with history of craniopharyngioma status post resection in 2007, panhypopituitarism on hormonal replacement, and obstructive hydrocephalus status post ventriculoperitoneal (VP) shunt, who presented with recurrent episodes of hyponatremia requiring hospitalization. During the clinical course of the disease since 2007, the patient was on hormonal replacement with hydrocortisone for hypocortisolism and desmopressin for diabetes insipidus with recommendations for stress dose of steroids during episodes of illnesses. Treatment with desmopressin and adrenal insufficiency as well as non-compliance with stress dose steroids were the compounding factors, causing severe hyponatremia. This case illustrates the importance of close monitoring of sodium levels during treatment with desmopressin as well as optimum steroid replacement in managing recurrent severe hyponatremia.

## Introduction

Craniopharyngioma is a benign epithelial tumor that typically arise in the suprasellar area of the brain, extending to involve the hypothalamus, optic chiasm, cranial nerves, third ventricle, and major blood vessels. It can produce a wide array of symptoms such as headaches, nausea, and vomiting, as well as visual and endocrine disturbances. Multiple hormonal deficiencies (adrenocorticotropic hormone (ACTH), thyroid-stimulating hormone (TSH), luteinizing hormone (LH), follicular stimulating hormone (FSH), growth hormone (GH)) due to dysfunction of anterior pituitary as well as antidiuretic hormone (ADH) deficiency due to involvement of posterior pituitary occur [1]. The treatment for craniopharyngiomas is challenging because of its location, invasiveness, and proximity to adjacent neurovascular structures. Multiple modalities can be implemented in the management of craniopharyngioma including surgery, radiotherapy and intracystic therapy [2]. Resection of craniopharyngiomas causes diabetes insipidus due to ADH deficiency, which requires treatment with desmopressin, in addition to various other hormonal deficiencies that require hormonal replacement. This case demonstrates a challenge in managing a patient with recurrent symptomatic severe hyponatremia on desmopressin after craniopharyngioma resection.

## Case presentation

This is case presentation of a 20-year-old male with history of craniopharyngioma status post resection in 2007, panhypopituitarism on hormonal replacement, obstructive hydrocephalus status post ventriculoperitoneal (VP) shunt, and recurrent episodes of hyponatremia, who was referred from outside hospital to the ICU at our facility due to altered mental status, nausea, vomiting, poor appetite, and hyponatremia. He was on hydrocortisone replacement therapy with stress dose steroids during concurrent illnesses as well as levothyroxine but desmpopressin was being held for two years. Workup revealed severe hyponatremia with sodium 121 mEq/L, low serum osmolality, and appropriately increased urine osmolality; urine sodium was greater than 40 mmol/L (Table 1). Hypertonic saline was started and stress dose of steroids followed by taper was given. Sodium levels improved gradually, hypertonic saline was discontinued, and oral intake encouraged along with steroids. He was initially given hydrocortisone 50 mg IV every six hours for 24 hours, then transitioned to oral hydrocortisone 40 mg three times daily for three days, 40 mg twice daily for another three days, 30 mg twice daily for three days and 20 mg twice daily thereafter.

**Table 1:** Lab values during current presentation

Lab	Value
Serum sodium	121 mEq/L (normal range 133-144 mEq/L)
Serum osmolality	260 mOsm/kg (normal range 282-305 mOsm/kg)
Urine osmolality	287 mOsm/kg (normal range 50-1200 mOsm/kg)
Urine sodium	45 mmol/L (normal range 40-220 mmol/L)

Following is the clinical course of the patient. In 2007, he presented with acute obstructive hydrocephalus secondary to craniopharyngioma and bilateral vision impairment left more than the right. Left frontotemporal craniotomy for gross total resection of the craniopharyngioma was done in addition to placement of an external ventricular drain. Bilateral optic atrophy was noted. Post operatively, ACTH deficiency, central hypothyroidism, diabetes insipidus, and GH deficiency was noted, and the patient was started on levothyroxine, hydrocortisone replacement, and desmopressin. 

Patient was lost to follow-up from endocrinology for three years and presented in 2015 with seizures and lethargy. Labs showed severe hyponatremia with serum sodium of 120 mEq/L, low serum osmolality, low urine osmolality, as well as low urine sodium (Table 2).

**Table 2:** Lab values in 2015 when patient presented after being lost to follow-up

Lab	Value
Serum sodium	120 mEq/L (normal range 133-144 mEq/L)
Serum osmolality	268 mOsm/kg (normal range 282-305 mOsm/kg)
Urine osmolality	35 mOsm/kg (normal range 50-1200 mOsm/kg)
Urine sodium	<10 mmol/L (normal range 40-220 mmol/L)

He recovered with hypertonic saline and stress dose of hydrocortisone. According to his parents, the patient had been receiving his desmopressin and his levothyroxine but was not getting his stress dose of hydrocortisone. The patient was advised to continue hydrocortisone 3.75 mg twice daily and stress dose of hydrocortisone 5 mg three times daily for the duration of illness. For more severe stresses, he was advised to increase the dose to 7.5 mg three times daily. For severe distress, such as lethargy or seizures, hydrocortisone 50 mg intramuscular was recommended. Levothyroxine was continued at 44 mcg daily. Desmopressin dose was 0.012 mg in the morning and 0.0375 mg in the afternoon. GH treatment was offered, but the parents declined it. Later that year, lab values showed his sodium in low 130s, low serum osmolality, and low urine osmolality and sodium levels (Table 3). 

**Table 3:** Lab values after which desmopressin was continued only in the evening

Lab	Value
Serum sodium	132 mEq/L (normal range 133-144 mEq/L)
Serum osmolality	264 mOsm/kg (normal range 282-305 mOsm/kg)
Urine osmolality	81 mOsm/kg (normal range 50-1200 mOsm/kg)
Urine sodium	16 mmol/L (normal range 40-220 mmol/L)

In 2017, patient was hospitalized for hyponatremia occurring with a concurrent diarrheal illness. Stress dose hydrocortisone was given and desmopressin was held until the sodium levels normalized. IV normal saline was administered to treat hyponatremia likely due to volume depletion. A similar picture due to fever and throat infection was noted in 2018, and similar management as previous was done. Usual dose of hydrocortisone was increased to 5 mg in the morning and 5 mg in the evening. Stress dose of hydrocortisone was increased to 7.5 mg three times daily for the duration of illness and for severe illness, 10 mg three times daily was recommended. Later that year, an episode of hyponatremia occurred again due to the patient missing his dose of hydrocortisone. Workup revealed severe hyponatremia with sodium 116 mEq/L, low serum osmolality, increased urine osmolality, and increased urine sodium from prior (Table 4). He was treated with hypertonic saline and parents were insisted on the need for compliance with hydrocortisone and the need to hold desmopressin during illnesses to prevent hyponatremia. 

**Table 4:** Lab values in 2017 during episode of non-compliance to stress dose of steroids

Lab	Value
Serum sodium	116 mEq/L (normal range 133-144 mEq/L)
Serum osmolality	247 mOsm/kg (normal range 282-305 mOsm/kg)
Urine osmolality	274 mOsm/kg (normal range 50-1200 mOsm/kg)
Urine sodium	42 mmol/L (normal range 40-220 mmol/L)

**Figure 1:**
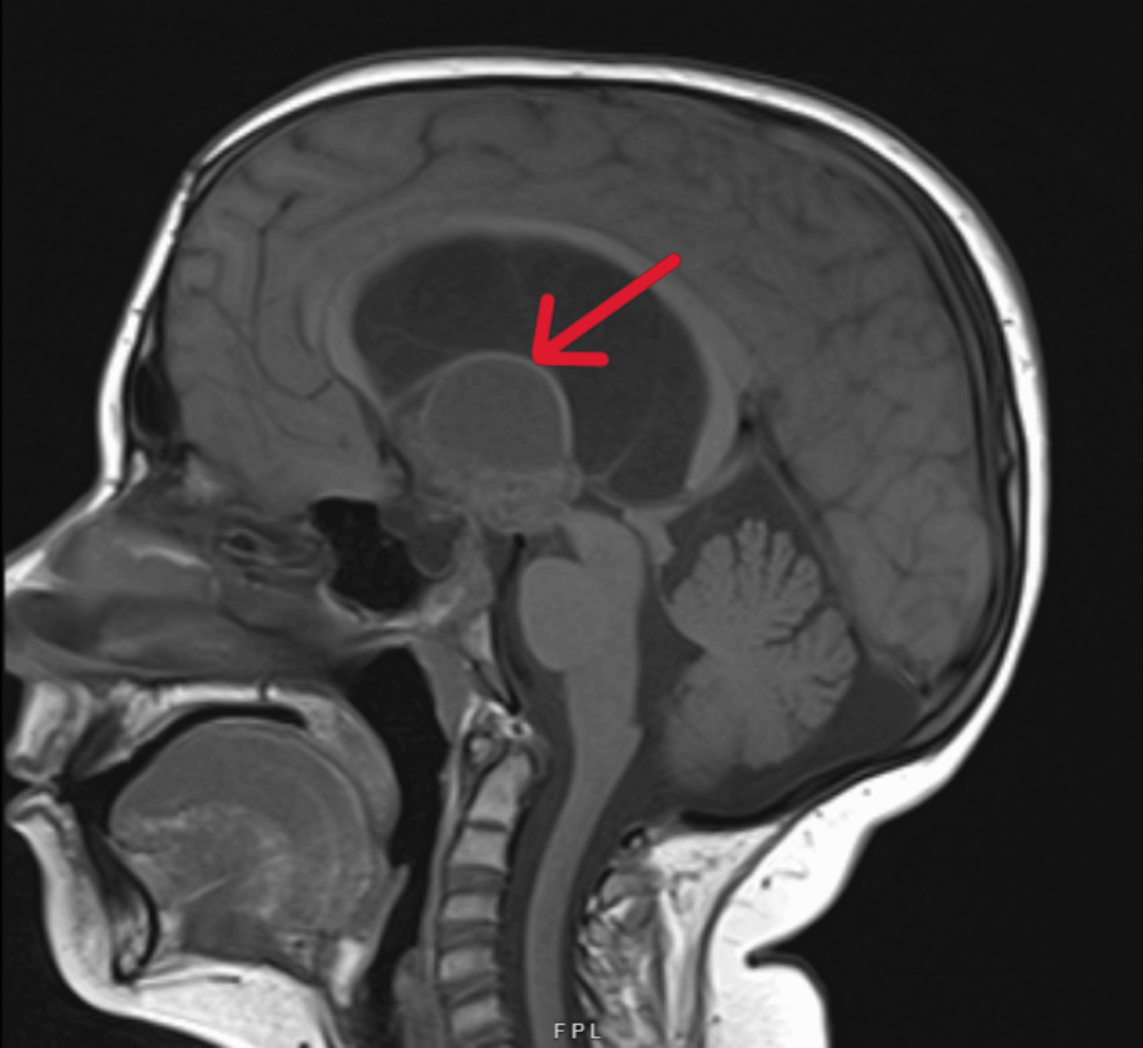
MRI brain with and without contrast, 2022 The arrow depicts craniopharyngioma comprising of multiple cystic components and a solid nodular enhancing component extending into the hypothalamus as well as along the third ventricle, causing compression of the foramen of Monro and hydrocephalus. The largest cystic component measured approximately 2.8 cm. The nodular enhancing lobular area along the inferior aspect of the mass measured 2.9 cm.

**Figure 2:**
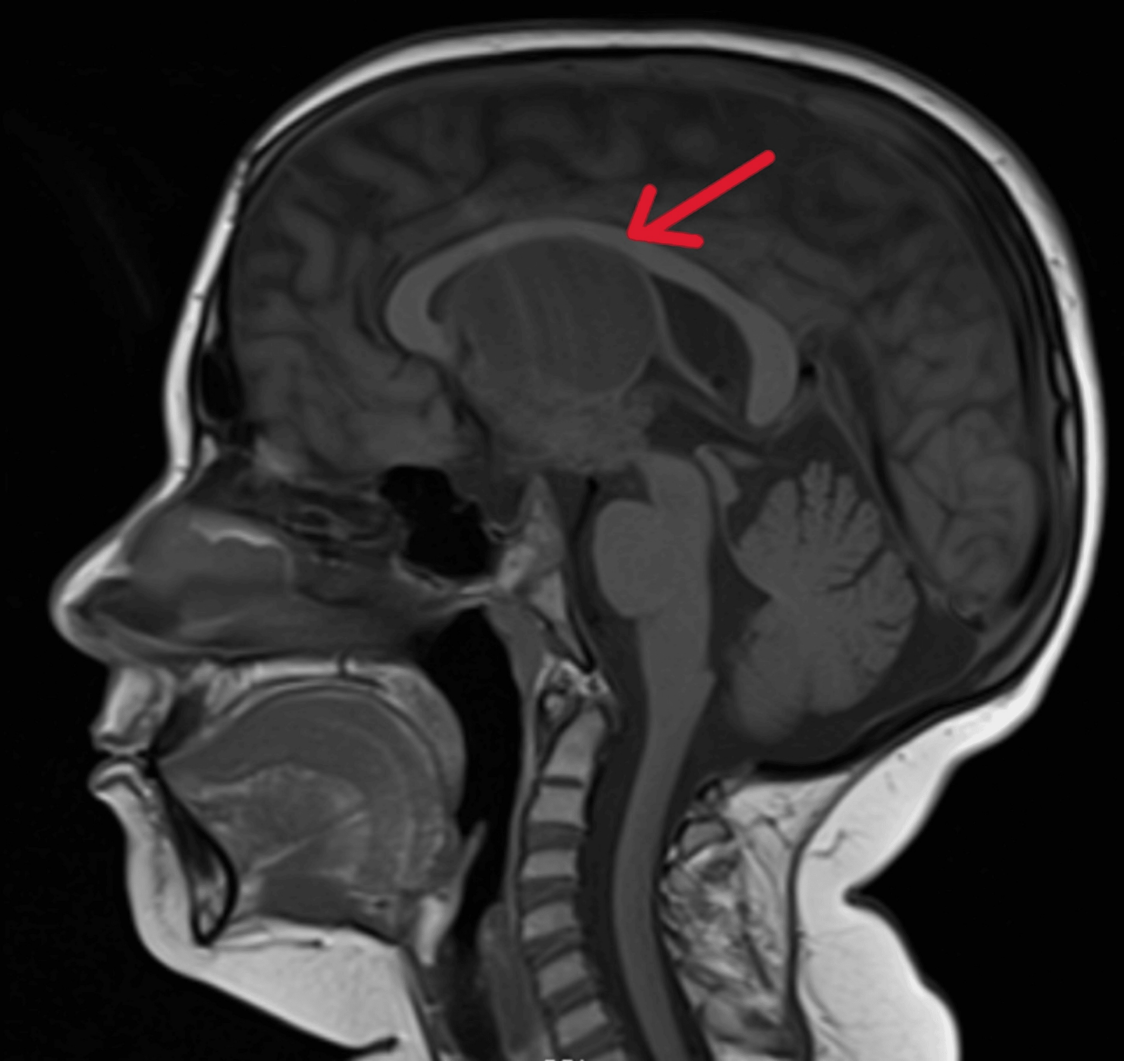
MRI brain with and without contrast, 2023 The arrow depicts craniopharyngioma with a large midline cyst extending into the ventricles which measured approximately 5.5 x 3.5 x 3.5 cm and was almost double compared to the preoperative MRI from September 2022. Two paramedian cysts also significantly increased in size, measuring approximately 1.5 cm each.

**Figure 3:**
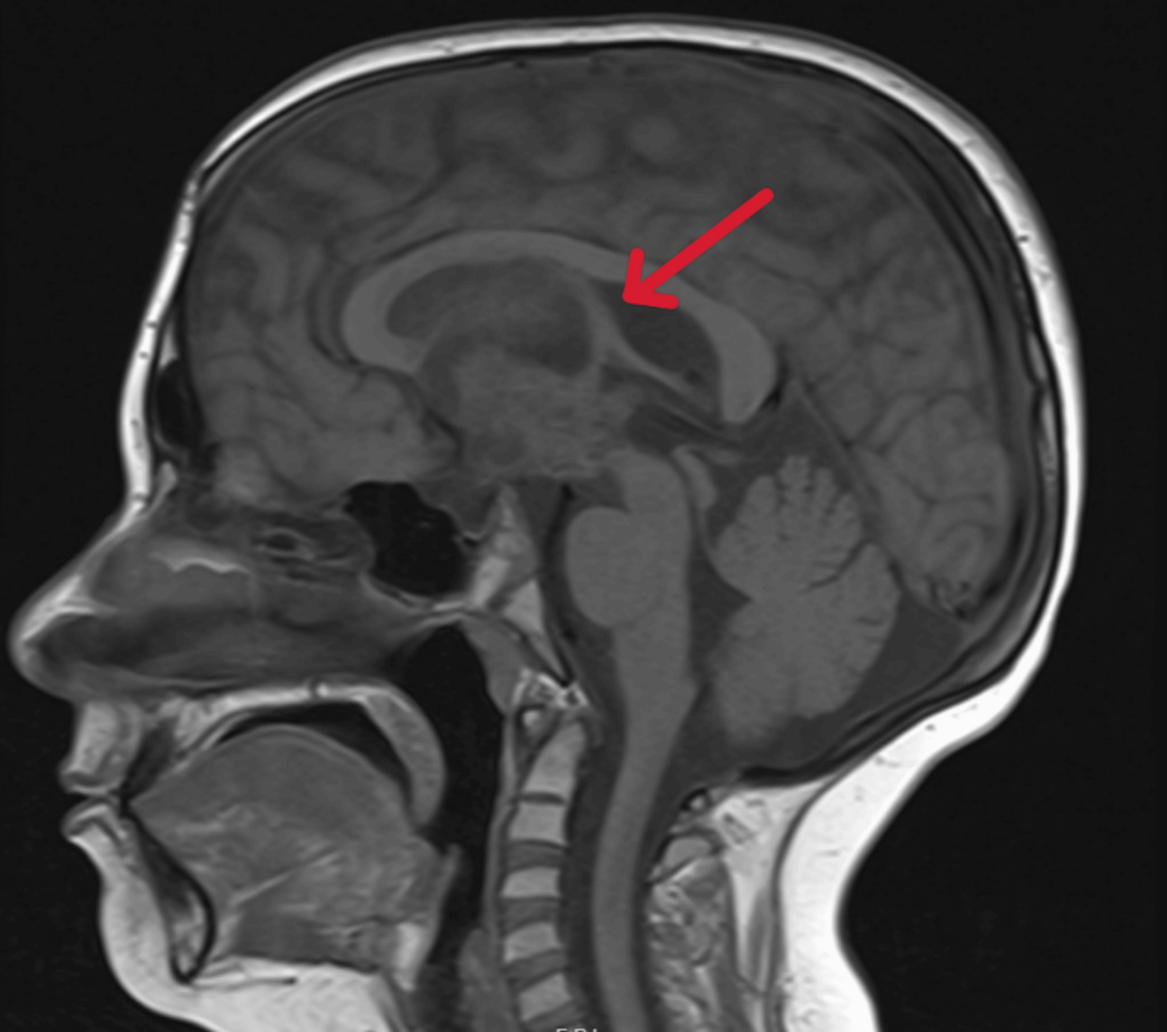
MRI brain with and without contrast, 2024 The arrow depicts craniopharyngioma with enlarged solid and cystic components. Heterogenous solid component of the mass extended more posteriorly involving hypothalamus region protruding into the third ventricle and extending towards the interpeduncular cistern. Dorsally it extended and completely obstructed the foramen of Monro. The solid component of the mass approximately measured 3.2 x 2.5 cm.

Over the years, patient's suprasellar mass consisting of both cystic and solid components continued to increase in size (Figure 1) and caused hydrocephalus, for which VP shunt placement was done in 2022. Levothyroxine was increased to 62.5 mcg daily and hydrocortisone to 5 mg twice daily. In 2023, the size of craniopharyngioma (Figure 2) continued to increase and further interval progression with enlarged solid and cystic components was noted in 2024 (Figure 3). 

Surgical intervention was deemed necessary but declined by the patient and the parents. Also, due to multiple hospitalizations for hyponatremia and no severe symptoms of diabetes insipidus such as polyuria, the patient was advised to stop desmopressin and liberalize fluid intake.

## Discussion

Resection of craniopharyngioma causes diabetes insipidus (ADH deficiency) and hypocortisolism (ACTH deficiency) in addition to various other hormonal deficiencies [3].

Diabetes insipidus caused by ADH deficiency is characterized by loss of free water and inability to concentrate the urine. ADH synthesis occurs in the hypothalamus and these storage vesicles are transported down the neuron’s axon through the hypothalamic-hypophysial tract, where they are ultimately released in the posterior pituitary. ADH principally exerts its effects by binding to the kidneys principal cells within the late distal tubule and collecting ducts. ADH binds to the V receptor on these cells and leads to the activation of adenylate cyclase, which causes a subsequent increase in the second messenger cyclic adenosine monophosphate (cAMP) which in turn initiates an intracellular phosphorylation cascade. Ultimately, intracellular aquaporin-2 (AQP2) storage vesicles are phosphorylated, which promotes their movement and insertion into the apical membrane. AQP2 is a water channel that allows water to move passively into the cell, thus promotes reabsorption of water in the kidney [4].

The most common presenting signs of diabetes insipidus include polydipsia, polyuria, and nocturia. Labs reveal hypernatremia with serum sodium levels >145 mEq/L and serum osmolality >295 mOsm/kg. Hypovolemia, low urine osmolality (<200 mOsm/kg), and decreased urinary sodium level are also seen [5]. Diabetes insipidus is treated by desmopressin, which is a selective vasopressin and A2 receptor agonist that helps with the reabsorption of water through the collecting duct and distal convoluted tubules of the kidneys [6]. However, a major side effect of desmopressin is hyponatremia, which requires close monitoring [7].

ACTH deficiency leading to hypocortisolism also causes hyponatremia. Cortisol causes direct suppression of ADH secretion and its deficiency results in increased ADH levels, free water retention, and hyponatremia [8]. Laboratory findings of hyponatremia due to cortisol deficiency reveal low serum osmolality, increased urinary sodium excretion, and increased urine osmolality. Patients with adrenal insufficiency secondary to craniopharyngioma resection need to be strictly compliant with stress doses of steroids during illnesses to prevent hyponatremia due to glucocorticoid deficiency.

Our patient was treated with desmopressin for diabetes insipidus in addition to corticosteroids for adrenal insufficiency. The early part of clinical course workup showed low serum sodium levels, low serum osmolality, low urine osmolality, and low urinary sodium excretion. The most likely cause was a mixed picture caused by diabetes insipidus superimposed on cortisol deficiency due to non compliance with stress dose steroids. Therefore, he continued to be treated with desmopressin in addition to emphasis on stress dose steroids during episodes of illnesses to prevent hyponatremia. Over the clinical course, the patient's labs showed uptrending urine osmolality and urine sodium excretion from prior. Since the patient was having multiple episodes of hyponatremia and no bothersome symptoms of diabetes insipidus such as polyuria/nocturia, and lab work was more suggestive of hyponatremia due to cortisol deficiency, desmopressin was decreased and finally stopped.

This case demonstrates the need to consider both desmopressin used for the treatment of diabetes insipidus as well as cortisol deficiency in managing hyponatremia after resection of craniopharyngioma. There can be an overlap based on lab values as seen in this patient who required both desmopressin as well as steroid treatment. Compliance with stress dose of steroids is necessary during periods of illnesses to prevent hyponatremia due to cortisol deficiency. Also, providers need to be wary of hyponatremia caused by desmopressin, and titration of its dose is necessary based on sodium levels as well as the clinical picture of the patient such as symptoms of polyuria/nocturia.

## Conclusions

This is a challenging case of management of recurrent hyponatremia seen after craniopharyngioma resection. Desmopressin, used for the treatment of diabetes insipidus, needs close monitoring of sodium levels to prevent hyponatremia. Also, there can be an overlap between cortisol deficiency, causing hyponatremia that requires treatment, especially in periods of illnesses, and stress dose of steroids.
